# Mid-Infrared Sensor Based on Dirac Semimetal Coupling Structure

**DOI:** 10.3390/s22062116

**Published:** 2022-03-09

**Authors:** Yuxiao Zou, Ying Liu, Guofeng Song

**Affiliations:** 1 Institute of Semiconductors, Chinese Academy of Sciences, Beijing 100083, China; zouyuxiao@semi.ac.cn; 2 College of Materials Science and Opto-Electronic Technology, University of Chinese Academy of Sciences, Beijing 100049, China; 3 College of Chemistry, Beijing Normal University, Beijing 100875, China; 11112017013@bnu.edu.cn

**Keywords:** Dirac semimetal, Goos–Hänchen shift, LRSPR, SPP, mid-infrared, biochemical sensor

## Abstract

A multilayer structure based on Dirac semimetals is investigated, where long-range surface plasmon resonance (LRSPR) of a dielectric layer/Dirac semimetal/dielectric layer are coupled with surface plasmon polaritons (SPPs) on graphene to substantially improve the Goos–Hänchen (GH) shift of Dirac semimetals in the mid-infrared band. This has important implications for the study of mid-infrared sensors. We studied the reflection coefficient and phase of this multilayer structure using a generalized transport matrix. We established that subtle changes in the refractive index of the sensing medium and the Fermi energy of the Dirac semimetal significantly affected the GH shift. Our numerical simulations show that the sensitivity of the coupling structure is more than $2.7\times 10^{7}\;\lambda /RIU$, which can be used as a potential new sensor application. The novelty of this work is the design of a tunable, highly sensitive, and simple structured mid-infrared sensor that takes advantage of the excellent properties of Dirac semimetals.

## 1. Introduction

In condensed matter physics, topological materials have received much research interest because of their ability to contain relativistic fermions with low-energy excitations [1,2,3,4]. Dirac semimetals (DSMs) are a type of topological material, famous for their topologically protected linear dispersive energy bands. Unlike the first studied two-dimensional (2D) Dirac systems, where the graphene energy band crossings are susceptible to perturbation, the system perturbation can only slightly move the three-dimensional (3D) Dirac cone of the DSM without eliminating it, which is quite robust [5]. This unique energy band structure endows the DSM with distinctive properties that make it suitable for optoelectronic applications [6,7,8,9]. Since their theoretical prediction, numerous angle-resolved photoemission spectroscopy and scanning tunneling microscope experiments have confirmed the existence of conical features in the energy band structure [10,11,12], giving rise to a large number of subsequent theoretical and experimental follow-ups that have enriched the understanding of DSMs.

Dirac semimetals are not only scientifically attractive, but they also have many potential applications. Owing to their energy-gapless tapered energy band, they have important potential applications in high-speed, broadband optoelectronic devices owing to their ultra-high mobility and broadband optical absorption [10,13,14,15]. The mid-infrared band (2–20 μm) is widely favored for scientific research due to its extremely high utility [16,17,18,19]. However, due to material limitations, it has been relatively little studied compared to the visible band. Additionally, the semi-metallic nature of DSM makes it highly promising. In the mid-infrared to terahertz band, DSM and graphene have a dielectric constant below zero, which can excite surface plasmon resonance (SPR) with less loss than conventional metals (e.g., Ag and Au) and is expected to be an excellent candidate for SPR [20,21]. Like graphene, its tunability is more prominent [22,23,24], and its thickness can be freely tailored to ensure stronger light-matter interactions. 

Goos–Hänchen (GH) shifts have attracted attention for numerous studies and applications in optics, chemistry, and biomedicine [25,26,27,28,29]. However, small shifts in ordinary structures have limited their research. Currently, large GH shifts have been obtained [30,31,32] by SPR, long-range surface plasmon resonance (LRSPR), etc. In the visible band, the coupling of two electromagnetic modes can further increase the GH shift and obtain a higher sensor sensitivity [33]. Therefore, the special structure may effectively improve the GH shifts.

In this paper, we propose a Dirac semimetal-based coupling structure. The large GH shift of this coupled structure in the mid-infrared band was studied based on the generalized transfer matrix. This large GH shift can be regulated by the thickness of the coupling layer, thickness of the Dirac semimetal, and Fermi energy. We also analyzed the effect of the refractive index of the sensing medium on the GH effect, showing excellent sensing performance.

## 2. Theoretical Model and Method

**Figure 1:**
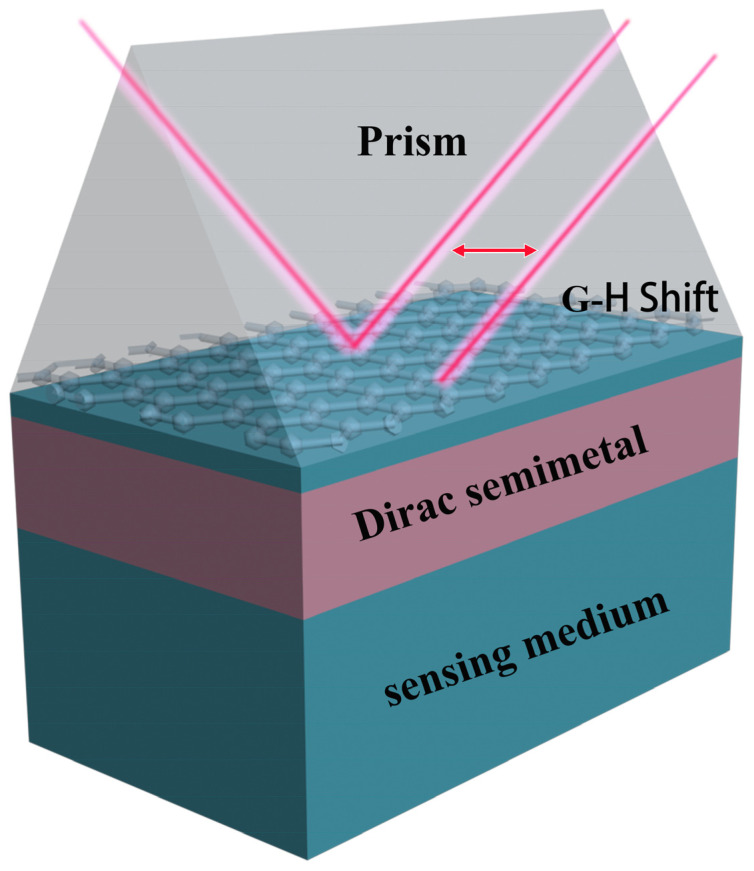
Schematic of the proposed structure that enhanced the GH shift, which contains layers of prism, graphene, sensing medium, and Dirac semimetal.

We propose a coupling structure to improve and control GH shifts, as depicted in Figure 1. Graphene is under the prism, and the refractive index of the prism is $n_{1}=2$, which is used to increase the wave vector. The second and fourth layers are the sensing medium ($n_{2}=1.35$) [34,35,36,37], and the DSM is fitted between the sensing medium. In brief, our structure can be considered as a common SPR sensor with a thin layer of Dirac semimetal immersed in the analyte solution. Graphene can be transferred to the prism with the assistance of PDMS [38].

First, we verified the SPP at the graphene/sensing medium interface. The surface conductivity of graphene can be described by the well-known Kubo formula, for $k_{B}T\ll E_{f}$ [39,40,41]:

(1) $$\sigma_{\mathrm{GR}}\left(\omega ,E_{f}\right)=\frac{ie^{2}}{4\pi \hbar }\ln\left[\frac{2\left|E_{f}\right|-\left(\omega +i2\Gamma \right)\hbar }{2\left|E_{f}\right|+\left(\omega +i2\Gamma \right)\hbar }\right]+\frac{ie^{2}k_{B}T}{\pi \hbar^{2}\left(\omega +i2\Gamma \right)}\left[\frac{E_{f}}{k_{B}T}+2\ln\left(e^{-\frac{E_{f}}{k_{B}T}}+1\right)\right]$$

where *e* is elemental charge, ħ and ω represent the reduced Planck constant and angular frequency of incident light, respectively; $E_{f}$ is the fermi energy, *T* is the temperature, and Γ is a phenomenological scattering rate ($E_{f}=0.35\;\mathrm{eV},\;T=300\;K,\;\Gamma =0.1\;\mathrm{meV}$). In the mid-infrared band, the dielectric constant of graphene is below zero, therefore it can excite SPPs instead of metals. The SPP dispersion relationship excited by the graphene/sensing medium can be expressed by the following formula [42]:

(2) $$k_{spp}=k_{0}\sqrt{\frac{\varepsilon_{graphene}\varepsilon_{sensing}}{\varepsilon_{graphene}+\varepsilon_{sensing}}}$$

The effective dielectric constant can be defined as $n_{eff}=k_{spp}/k_{0}$.

Previous studies have shown that the conductivity of the DSM can be described by the Kubo formula [21,43]. For $k_{B}T\ll E_{f}\;$and long-wavelength limit, the optical conductivity can be written analytically as

(3a) Reσ(Ω )=e2ħ2gEf24πvfΩΘ(Ω−2)

(3b) Imσ(Ω)=e2ħ2gEf24π2vf[4Ω−Ωln(4εc2|Ω2−4|)]

where *g* is the degeneracy factor, $v_{f}$ is the Fermi velocity, Θ(x) is the step function, and $\varepsilon_{c}$ represents the high-energy cutoff of the linear model. Here, Ω is the normalized frequency $\hbar \omega /E_{f}$.

To accurately describe the dielectric function of the DSM, a two-band model considering inter-band electron transition is used instead of a simple Drude-like model [43]:

(4) $$\varepsilon_{DSM}=\varepsilon_{b}+\frac{i\sigma }{\omega \varepsilon_{0}}$$

**Figure 2:**
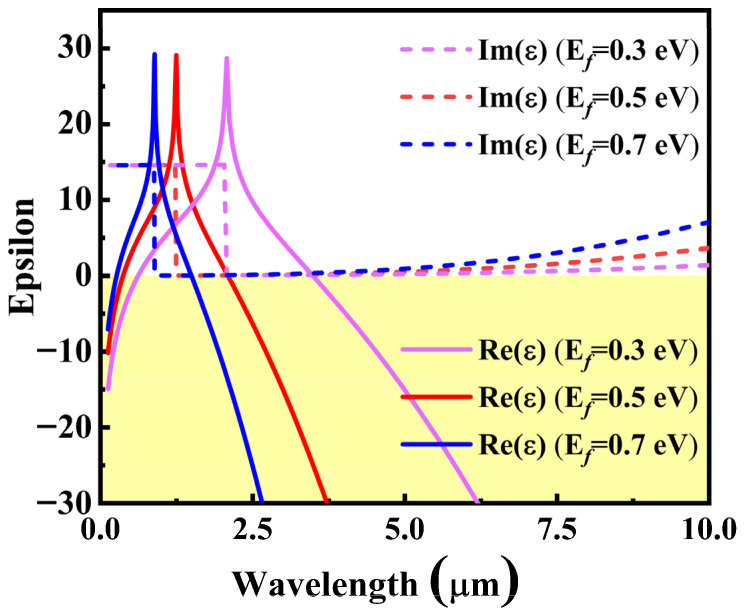
Representative behavior of the DSM dielectric functions. The solid line and the dash line represent the real and imaginary parts of the DSM, respectively. The parameters are set as $\varepsilon_{b}=1$, g=40, $\varepsilon_{c}=3$, $v_{f}=10^{6}\;m/s$, $\tau =4.5\times 10^{-13}\;s$.

Therefore, when considering the Drude damping ($\Omega +i\hbar /\tau E_{f}\;$ instead of Ω), the dielectric function of the DSM is shown in Figure 2. 

In the middle infrared band, the real part of the dielectric function of the DSM is below zero and has a suitable imaginary part, which indicates a good SPP material [42]. In the case of being fitted by the sensing medium, an insulator–metal–insulator (IMI) structure can be formed to excite the LRSPR. Through the boundary conditions of the symmetrical IMI structure [42], the dispersion relationship can be derived as

(5) $$tanh\;k_{1}a=-\frac{k_{1}\varepsilon_{2}}{k_{2}\varepsilon_{1}}$$

where, $k_{i}^{2}=k_{spp}{}^{2}-k_{0}^{2}\varepsilon_{i}$,i=1,2.

For this coupling structure, we used a generalized transfer matrix [44] to calculate the reflectivity and phase at TM incidence. This method can deal with different types of materials arbitrarily, which does not exist in discontinuous solutions.

For multilayer systems, using the electromagnetic field boundary conditions, we can connect the electromagnetic wave amplitudes of two adjacent layers [44]:

(6) $$A_{i-1}{\vec{E}}_{i-1}=A_{i}{\vec{E}}_{i}$$

where $A_{i}$ is the dynamic matrix with respect to the electric field vector $\gamma_{i}$, and i is the *i*th layer.

To better define the transmission matrix, the propagation matrix is introduced by **P** [44,45]:

(7) $$P_{i}=\left(\begin{array}{cccc} e^{-i\frac{\omega }{c}q_{i1}d_{i}} & 0 & 0 & 0\\ 0 & e^{-i\frac{\omega }{c}q_{i2}d_{i}} & 0 & 0\\ 0 & 0 & e^{-i\frac{\omega }{c}q_{i3}d_{i}} & 0\\ 0 & 0 & 0 & e^{-i\frac{i}{c}q_{i4}d_{i}} \end{array}\right)$$

where c is the speed of light, ω is the angular frequency of the incident light, and $q_{ij}\;\left(d_{i}\right)$ are the four eigensolutions (j=1, 2, 3, 4) of the z-component of the wave vector (thickness) in the ith layer of the material.

Thus, the transmission matrix of the whole structure can be written as

(8) $$Q=A_{1}^{-1}A_{2}P_{2}A_{2}^{-1}\ldots A_{i-1}P_{i-1}A_{i-1}^{-1}A_{i}$$

Based on the transmission matrix **Q**, the reflection coefficient of the TM polarization incidence can be calculated as follows [44,45]:

(9a) $$r_{pp}=\frac{Q_{11}Q_{43}-Q_{41}Q_{13}}{Q_{11}Q_{33}-Q_{13}Q_{31}}$$

(9b) $$r_{ps}=\frac{Q_{41}Q_{33}-Q_{43}Q_{31}}{Q_{11}Q_{33}-Q_{13}Q_{31}}$$

Here, the subscripts *pp* and *ps* represent the *p* and *s* reflections of *p* incidence, respectively.

After obtaining the reflection coefficient, according to the static phase method, the GH shift can be expressed as

(10) $$D_{GH}\left(\theta ,\omega \right)=-\frac{\lambda }{2\pi }\frac{d\phi_{r}}{d\theta }\;=-\frac{\lambda }{2\pi }\left(Re\left(r\right)\frac{dIm\left(r\right)}{d\theta }-Im\left(r\right)\frac{dRe\left(r\right)}{d\theta }\right)\;$$

Here, θ is the angle of incidence, λ is the wavelength, and $\phi_{r}$ is the reflected phase.

As $r_{ps}$ is proportional to the off-diagonal components’ dielectric function, all the materials in our structure $\varepsilon_{xy}=-\varepsilon_{yx}=0$ without considering the magnetic field effect. Therefore, the effect of $r_{ps}$ on GH shifts could be disregarded. In the following discussion, if not otherwise specified, we set the incident light wavelength to be *λ* = 2.5 µm.

Sensitivity is an important parameter for precision probing, meaning the degree of change in the amount of response is due to a change in the measured quantity. As the structure is sensitive to many parameters, a variety of sensitivities can be defined. For example, the change in the angle of the GH shifts maximum with respect to the Fermi energy can be defined and used to measure the ability to detect the Fermi energy level:

(11) $$S_{f}=\frac{d\theta }{dE_{f}}$$

Similarly, it can also be used as a refractive index sensor, and the ratio of the change in GH shifts caused by the change in refractive index of the sensing medium to the change in refractive index is used as the sensitivity:

(12) $$S_{n}=\frac{dGH}{dn}$$

## 3. Results and Discussion

**Figure 3:**
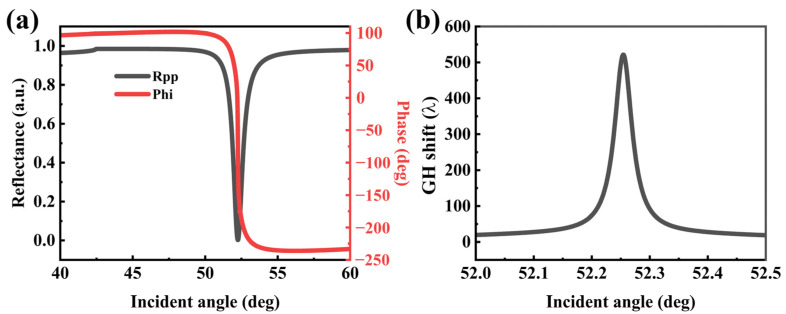
Variation in the (**a**) reflectance and phase, (**b**) GH shift for 350 nm DSM film with respect to the incident angle when $E_{f}$ = 0.5 eV, respectively. The other parameters of the DSM are the same as for Figure 2.

Figure 3a shows the reflection coefficient and phase with the angle of incidence. When $\theta_{SPR}$ = 52.255, a narrow peak in induced by the SPR. In the region of the peak, the reflection phase changes significantly. At this wavelength, the imaginary part of the DSM dielectric constant is very small; therefore, the reflection spectrum has a small half-width ($0.78^{0}$). According to Equation (10), acute phase changes within a narrow range results in large GH shifts. As shown in Figure 3b, a large GH shift of 521 *λ* is observed at the resonance peak.

**Figure 4:**
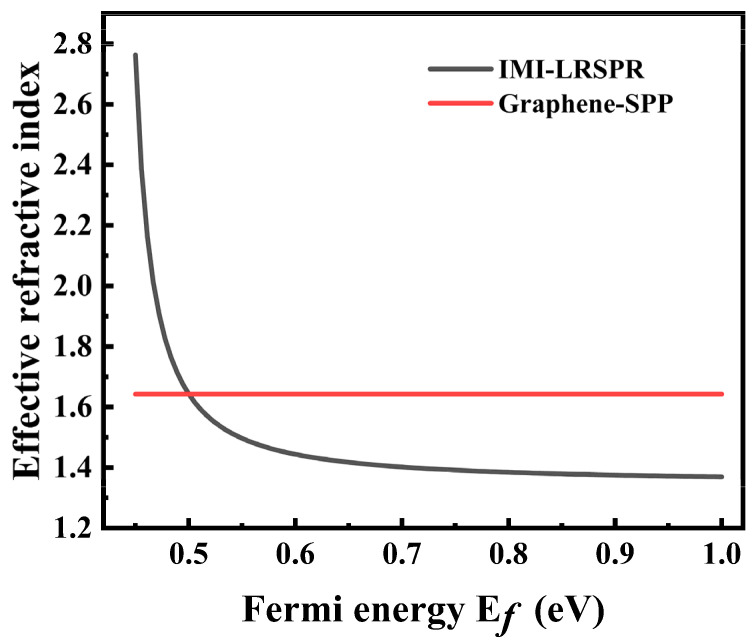
Effective refractive index of graphene-SPP and IMI-LRSPR as a function of $E_{f}$ of DSM.

Previous studies have reported that LRSPR can significantly reduce the half-peak width of the resonance, resulting in sharp phase changes and a significant increase in GH shifts [30]. To further increase the GH shifts, the structural coupling between graphene SPP and DSM LRSPR was used to obtain better performance. To verify that graphene SPPs can couple with LRSPR with an IMI structure, the coupling conditions were studied. When the incident light f = 119 THz, the effective refractive index varies with the $E_{f}$ of the DSM, as shown in Figure 4. 

Figure 4 shows that the effective refractive index of the IMI structure can be easily adjusted by $E_{f}$ of DSM, showing excellent adjustable performance. When the $E_{f}$ of the DSM is 0.5 eV, the two systems have the same effective refractive index (*n_eff_* = 1.64). In this case, the two modes can be coupled, which theoretically further increases the GH shift. 

**Figure 5:**
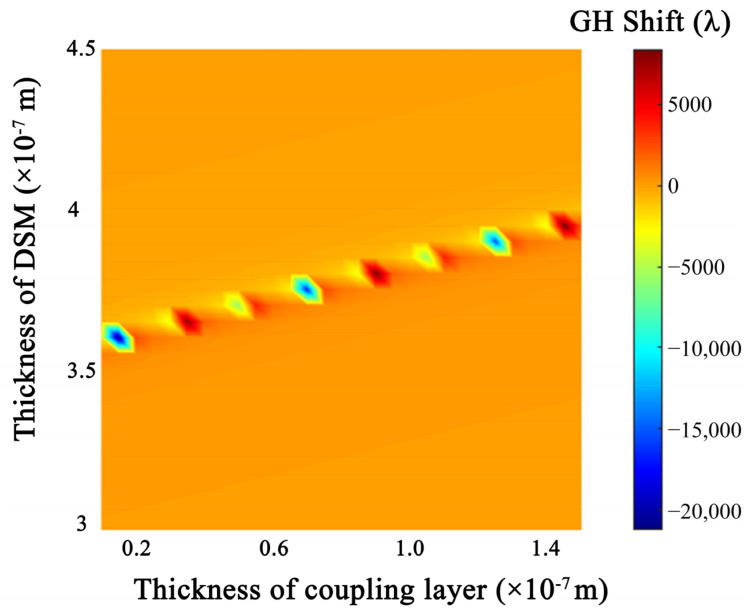
Dependence of the GH shifts on the optimizable structure parameters: the thickness of DSM and coupling layer.

In this coupled structure, the GH shifts are affected by many factors. For example, the thickness of the metal layer in the IMI structure that excites the LRSPR, the thickness of the coupling layer, etc. As shown in Figure 5, when the coupling layer thickness and DSM thickness are selected as the optimal thickness (e.g., d_DSM_ = 375 nm, dc = 70 nm), the phase change abruptly changes, resulting in a maximum GH shift.

**Figure 6:**
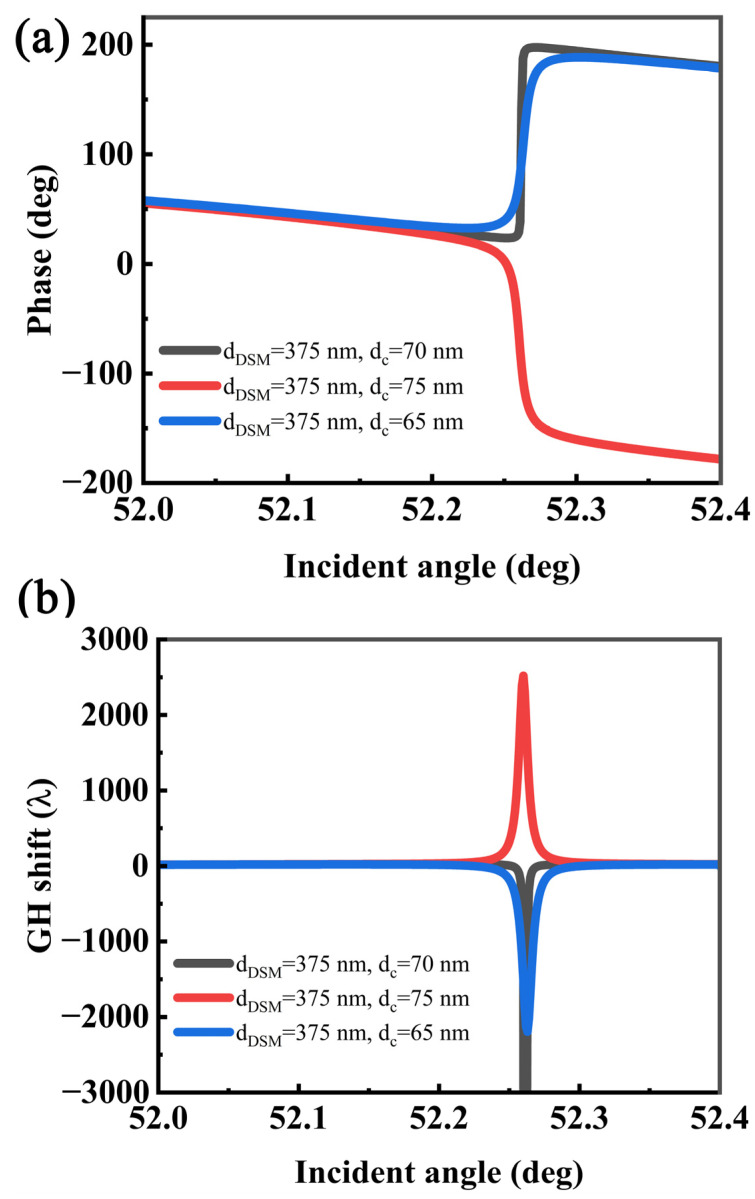
Calculated (**a**) phase and (**b**) GH shift as a function of the incident angle with different coupling layer thicknesses.

In this case, however, the phase loses its physical meaning and therefore cannot accurately describe the GH shifts [46,47]. It was found that when the thickness of the coupling layer is less than the optimal thickness, the phase decreases with the increase in the angle, and when the angle of resonance increases sharply, it results in negative GH shifts. When the thickness of the coupling layer is larger than the optimal thickness, the phase increases with the increase in the incident angle, resulting in positive GH shifts. Therefore, the direction of the GH shift can be precisely controlled by adjusting the thickness of the coupling layer without significantly changing the resonance angle. We can imagine that when the coupling layer is infinite, the two modes are isolated from each other and cannot be coupled, and the GH shifts is very small. As the thickness of the coupling layer decreases, the modes start to coupling and making the GH shifts larger. As the thickness of the coupling layer continues to decrease, the optimal coupling condition is lost and the GH shift decreases again, which is consistent with previous work [33]. On the other hand, the thickness of the insulator layer of the IMI structure also affects the GH shifts [47]. Even if it deviates from the optimal thickness (d_DSM_ = 375 nm, dc = 75 nm), the GH shift can still reach a large value of 2520 *λ*, which is equivalent to five times that of the ordinary DSM structure, as shown in Figure 6.

**Figure 7:**
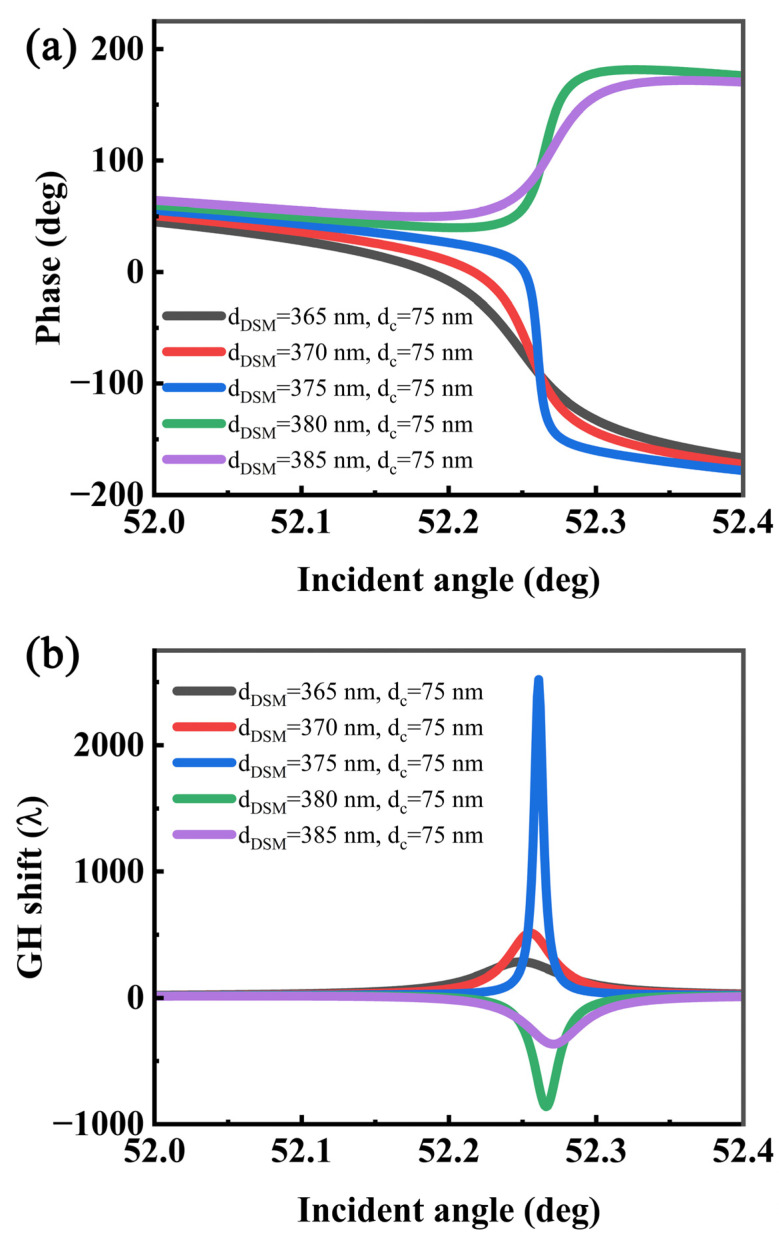
Relationship between the (**a**) phase, (**b**) GH shift, and angle with different DSM thicknesses.

Similarly, because the thickness of the DSM affects the LRSPR, the GH shifts can also be regulated by adjusting the thickness of the DSM, as shown in Figure 7. When the thickness of the coupling layer is at a non-optimal thickness, the thickness of the DSM can also significantly change the magnitude and direction of the GH and slightly change the angle of the GH shifts to a maximum. When the thickness of the DSM is less than 375 nm, the GH shift is positive and its reflection phase decreases with the increase in angle; when the thickness of the DSM is more than 375 nm, the GH shift is negative and the phase increases with the increase in angle. As the thickness of the DSM increases, the effective permittivity of its structure also increases, which causes the maximum value of the GH to shift slightly to a high angle. When the DSM thickness is far from the optimal value, its GH peak decreases significantly, and the half-peak width increases gradually owing to the gradual loss of resonance conditions.

**Figure 8:**
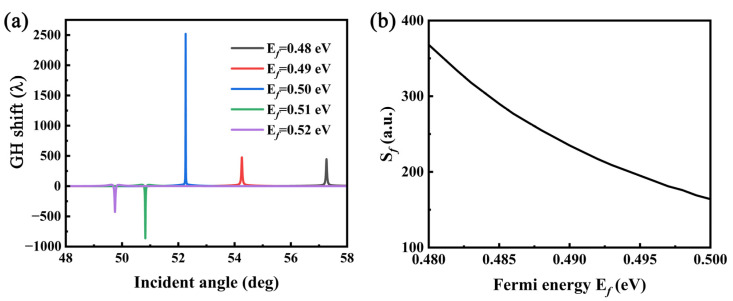
(**a**) Dependence of the GH shifts on the coupling structure at different $E_{f}$ for DSM. (**b**) Sensitivity as a function of $E_{f}$.

As the optical properties of Dirac semimetals are closely related to the Fermi energy, we can easily change the Fermi energy of Dirac semimetals by chemical doping, external gate electric field, etc. [48,49,50], to adjust the GH shift of the coupling structure. Similarly, this structure can not only adjust the positive and negative GH through the change in the DSM Fermi energy, but also adjust its resonance angle. This is mainly attributed to the fact that the Fermi energy significantly changes the permittivity of the DSM, which changes the effective refractive index of the structure. Additionally, the effective refractive index of the structure significantly affects the resonance angle [51]. As shown in Figure 8a, when the Fermi energy is less than 0.5 eV, the GH shift is negative, and when it is more than 0.5 eV, the GH shift is positive. We can define the Fermi energy sensitivity factor as $S_{f}$=$\;\frac{d\theta }{dE_{f}}$, and it is easy to detect subtle changes in the Fermi level, using the change in the resonance angle. When the Fermi level is changed from 0.48 eV to 0.52 eV, there is a clear change in the peak position, which has a sensitivity of more than 100 deg/eV in the range. It is easy to understand that as the Fermi level increases, the effective refractive index decreases, and its resonance angle decreases accordingly. It can be seen from Figure 4 that when the Fermi level is <0.5 eV, the effective refractive index of the IMI structure changes sharply, which can lead to a large change in the effective refractive index of the entire structure; therefore, it has a high sensitivity of more than 150°/eV in the range (in Figure 8b). 

**Figure 9:**
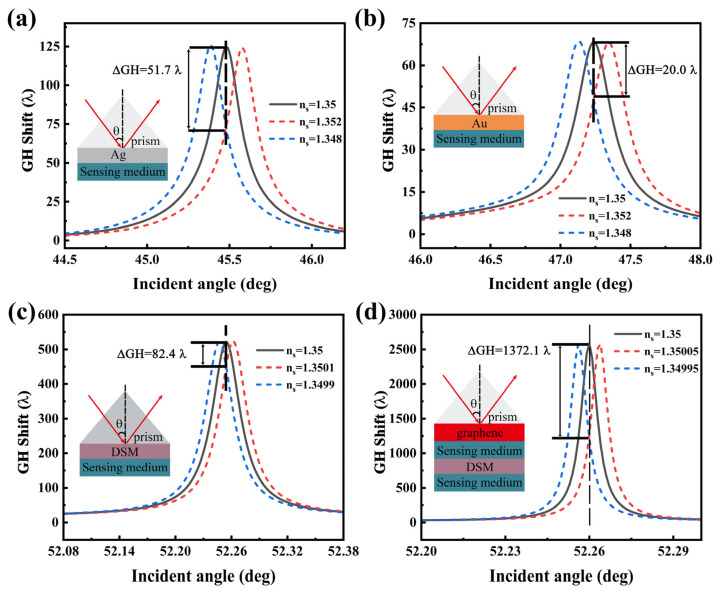
Dependence of the GH shifts with incident angle in a small refractive index range in (**a**,**b**) traditional metal Ag (Au) SPR structure (45 nm), (**c**) DSM based SPR structure (380 nm), and (**d**) DSM-based coupling structure.

Finally, we found that a slight change in the sensing medium results in a significant change in the GH displacement; therefore, the refractive index sensor can be designed accordingly. As shown in Figure 9, we compared it with the traditional metal SPR sensor and the SPR sensor of the DSM. In the traditional Au (Ag) sensor, we used an excitation wavelength of 632.8 nm, Au (Ag) film thickness of 45 nm, and n_Au_ = 0.7900 + 17.3109i, n_Ag_ = 0.1675 + 3.4728i. The parameters are consistent with previous studies [21,52,53]. When the refractive index of the sensing medium changes $\Delta_{n_{s}}=$ 0.002, the GH shifts changed by 51.7 λ (20.0 λ).According to Equation (12), the sensitivity can be obtained as *S_n_* $\approx 2.6\times 10^{4}\;\lambda \;\left(1\times 10^{4}\;\lambda \right)$. Additionally, based on the DSM structure, it has a larger ΔGH=82.4 λ while changing the smaller refractive index of the sensing medium ($\Delta_{n_{s}}=0.5\times 10^{-4}$). Therefore, it has a higher sensitivity, *S_n_*$\;\approx 1.6\times 10^{6}\;\lambda ,$ relative to the traditional metal sensor. The proposed coupling structure has large GH shifts compared to traditional metals. While the change in the sensing dielectric constant is very small, its GH shift changes sharply, with the purpose to obtain *S_n_* ≈ 2.7$\times 10^{7}\;\lambda$. Compared with traditional metal sensor, it has been improved by three orders of magnitude.

**Figure 10:**
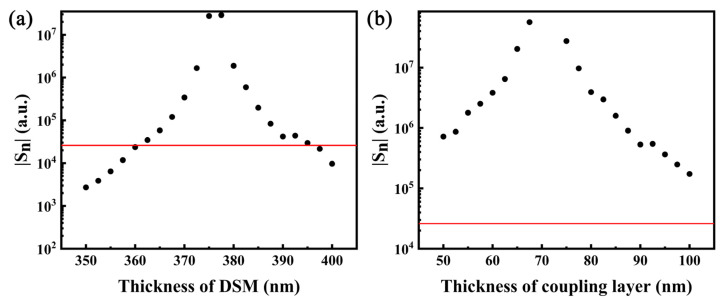
Variation in S_n_ with the thickness of (**a**) DSM (dc = 75 nm) and (**b**) coupling layer (d_DSM_ = 375 nm). The red line in these figures shows the sensitivity of the conventional metal Ag SPR structure as a comparison.

Figure 10 shows the variation in sensitivity S_n_ with the DSM thickness and coupling layer thickness. Its GH shift is the greatest near the optimum thickness, which also results in the greatest sensitivity. As one moves away from the optimum thickness, the sensitivity decreases; however, it is still is greater than that of the conventional metallic SPR structure within a certain range, which provides a process tolerance for the actual device.

Table 1 shows the GH shifts and sensitivities of the sensors with different materials, structures, and wavelengths. Our proposed structure shows significant improvement in both GH displacement and sensitivity, providing a potential solution for mid-infrared sensors.

## 4. Conclusions

In this paper, we proposed a coupling structure that can greatly enhance GH displacement. In this structure, the direction of the GH displacement can be controlled independently by adjusting the thickness of the coupling layer. As the angle of GH displacement is closely related to the Fermi energy of the DSM, the change in the Fermi level can be accurately measured by the change in the GH displacement angle, and the sensitivity of the Fermi level is up to. In addition, owing to the extremely large GH displacement of the coupling structure, it can be designed as an ultra-sensitive refractive index sensor with a sensitivity up to. We believe that this sensor, based on GH shifts, has the advantages of ultra-high sensitivity and adjustability, and has potential application prospects in precision measurement, biological monitoring, etc.

## Figures and Tables

**Table 1:** Comparison with the different materials SPR sensor.

Materials	Wavelength	GH Shift (λ)	Sensitivity (λ/RIU)	References
Au	632.8 nm	12.5	-	[54]
Cu-BlueP/WS2-graphene	632.8 nm	1004	$3.2\times 10^{6}\;$	[55]
Au-MoS2/graphene	632.8 nm	235.8	$5.5\times 10^{5}\;$	[56]
Au-ITO-MoS2/graphene	632.8 nm	801.7	$8.02\times 10^{5}\;$	[57]
Ag-Au-hBN-graphene	632.8 nm	182.1	$2.02\times 10^{5}\;$	[32]
DSM	8.9 um	361.4	-	[21]
Graphene-planar waveguide	10.6 um	less than −500	-	[58]
Graphene-photonic crystals	300 um	Less than −2000	-	[59]
Graphene-medium-DSM-medium	2.5 um	More than 2500	$2.7\times 10^{7}\;$	This work

## Data Availability

Not applicable.
